# Formation of PLGA–PEDOT: PSS Conductive Scaffolds by Supercritical Foaming

**DOI:** 10.3390/ma16062441

**Published:** 2023-03-18

**Authors:** Antonio Montes, Diego Valor, Yaiza Penabad, Manuel Domínguez, Clara Pereyra, Enrique Martínez de la Ossa

**Affiliations:** 1 Department of Chemical Engineering and Food Technology, Faculty of Sciences, University of Cadiz, International Excellence Agrifood Campus (CeiA3), 11510 Puerto Real, Cadiz, Spain; 2 Department Condensed Matter Physics and Institute of Electron Microscopy and Materials, Faculty of Sciences, University of Cadiz, International Excellence Agrifood Campus (CeiA3), 11510 Puerto Real, Cadiz, Spain

**Keywords:** supercritical foaming, conductive, polymer, porosity, PLGA

## Abstract

The usage of conjugated materials for the fabrication of foams intended to be used as therapeutic scaffolds is gaining relevance these days, as they hold certain properties that are not exhibited by other polymer types that have been regularly used until the present. Hence, this work aims to design a specific supercritical CO_2_ foaming process that would allow the production of porous polymeric devices with improved conductive properties, which would better simulate matrix extracellular conditions when used as therapeutic scaffolds (PLGA–PEDOT:PSS) systems. The effects of pressure, temperature, and contact time on the expansion factor, porosity, mechanical properties, and conductivity of the foam have been evaluated. The foams have been characterized by scanning electron and atomic force microscopies, liquid displacement, PBS degradation test, compression, and resistance to conductivity techniques. Values close to 40% porosity were obtained, with a uniform distribution of polymers on the surface and in the interior, expansion factors of up to 10 orders, and a wide range of conductivity values (2.2 × 10^−7^ to 1.0 × 10^−5^ S/cm) and mechanical properties (0.8 to 13.6 MPa Young’s modulus in compression test). The conductive and porous scaffolds that have been produced by supercritical CO_2_ in this study show an interesting potential for tissue engineering and for neural or cardiac tissue regeneration purposes due to the fact that electrical conductivity is a crucial factor for proper cell function and tissue development.

## 1. Introduction

Nowadays, composite 3D structures are increasingly gaining interest, particularly because of their proven ability to support cell attachment in in vitro cultures [1]. Flexibility and porosity are two of the most vital properties that these structures must exhibit if they are to be used for tissue engineering purposes. Therefore, polymers or polymer mixtures have been commonly used based on their advantageous mechanical properties [2]. To effectively mimic the behavior of extracellular matrices, these therapeutical devices must be made of materials with an internal structure formed by large voids or interconnected pores that facilitate the diffusion of oxygen and nutrients. These devices are commonly referred to as scaffolds [3].

Recent research studies have highlighted that biodegradable and conductive copolymers promote both cell proliferation and tissue repair [4,5,6]. Conductivity also plays a significant role with regard to supporting certain biological processes, such as the transmission of signals through the nervous system or the healing of damaged tissue. The potential benefits of using electrical stimulation to promote and enhance these processes is also a matter of increasing interest [7].

Conductive polymers have been demonstrated to have, either with or without electrical stimulation, a beneficial impact on cell proliferation and adhesion as well as high levels of biocompatibility [8,9]. Therapeutic scaffolds have been recently designed and made of conductive copolymers for their use in tissue engineering or as drug delivery systems that are stimulated by electrical signals. The most frequently used are polyaniline [10], polythiophenes [11], polypyrrole [12,13], or poly(2,3-dihydrothieno-1,4-dioxin) (PEDOT) [14]. In our work, we have investigated PEDOT:PSS (Poly(2,3-dihydrothieno-1,4-dioxin)–poly(styrenesulfonate)) scaffolds. To embed PEDOT:PSS into porous materials and to improve their mechanical properties through the addition of different aggregates [15], these types of scaffolds have been prepared in the literature using different techniques as follows: electrochemical polymerization surrounding a pre-synthesized scaffold [16]; vapor-phase polymerization for neural stem cell proliferation and differentiation [17]; or freeze-drying. The capacity of the body cells to attach to porous scaffolds plays a relevant role with regard to the impedance of polymeric networks and can, therefore, be used as a means to control cell growth. Compared to other polythiophene derivatives, PEDOT:PSS presents high chemical and thermal stability as well as a high and adjustable electrical conductivity [18]. Other advantages of PEDOT:PSS include its low oxidative strength, good visible light transparency, and high biocompatibility that makes it suitable for its usage with biodegradable polymeric scaffolds. PEDOT:PSS is commonly available in the form of an aqueous dispersion, which makes it appropriate for an array of coating methods [19].

Conductive polymers generally fail to possess some of the essential characteristics required for tissue engineering with regard to strength, flexibility, solubility, or durability. Furthermore, in some cases, when conductive polymers are in a liquid phase at room temperature, they require rather complex processing techniques [20]. In order to overcome these limitations, these polymers are often combined with or incorporated into other non-conductive polymeric structures. These structures provide the mechanical properties that are especially required, particularly for tissue repair. Poly(lactic-co-glycolic acid) (PLGA), polyvinylpyrrolidone (PVP), polylactide (PLA), polycaprolactone (PCL), polyvinyl acetate (PVAc), or polyvinyl alcohol (PVA) are some of the compounds often used for this purpose [21]. PLGA, in particular, is often preferred for this type of biomedical objective for a number of reasons, namely, due to its mechanical properties, its composition-dependent biodegradability, and its easy production by different methods [22,23,24]. PLGA also has exceptional processing capabilities that allow to produce different pore-size scaffolds that can be used in combination with other materials for the regeneration of a wide assortment of tissues. Its mechanical properties also make it a suitable choice for tissue regeneration treatments in terms of biocompatibility and degradability. Thus, PLGA’s chemical properties enable its hydrolytic degradation by deesterification, and its monomeric components are easily removed by natural means following its degradation. Nevertheless, and although PLGA’s degradation process may sometimes lead to a local acidic microenvironment when compared against other commonly used polymers, PLGA allows superior control over its degradation properties by modifying its monomers ratio (lactic:glycolic). In addition, the broad range of degradation rates exhibited by PLGA can be controlled by adjusting the composition of its chains, its crystallinity, and its hydrophobic/hydrophilic balance. It has been recently combined with other materials, such as ceramics, to enhance some of its properties and improve its performance in tissue repair treatments [25,26].

Currently, there are numerous techniques that have been commonly applied to generate porous scaffolds over the last decades, such as gas foaming [27], freeze drying [28], solvent-casting [29], 3D printing [30], electrospinning [31] or phase separation [32]. Nevertheless, on the one hand, the usage of high temperatures needs to be avoided in order to reduce the degradation that biodegradable polyesters may suffer over their processing. On the other hand, many of the above-mentioned processes are not capable of producing morphologically similar polymer matrices with a uniform pore size and a high level of interconnectivity between them [33]. 

Supercritical carbon dioxide (scCO_2_) foaming has emerged as a promising method to produce functional porous scaffolds that avoid some of the drawbacks associated with other methods, such as high temperatures, the usage of to organic solvents, or the poor homogeneity of the final structures [34]. CO_2_ is not only highly soluble in polymers, but it is also a non-toxic, inexpensive, and reusable compound that makes this a greener technique [35]. In its supercritical state, CO_2_ exhibits properties, such as density, diffusivity, and low viscosity, that enable it to permeate the polymeric matrix, resulting in the plasticization of the matrix by decreasing its glass transition temperature. After a controlled contact time, depressurization of the system leads to supersaturation, which induces cell nucleation during phase separation, ultimately resulting in the formation of the porous scaffold structure [36,37,38]. This approach requires that the polymer has a certain affinity with the CO_2_ phase. Therefore, this scaffold-producing technique is particularly suitable for its use with amorphous materials.

Several studies can be found in the literature that investigate the development of polymeric scaffolds produced by supercritical CO_2_ foaming. Thus, a variety of polymeric scaffolds were obtained via different approaches. Poly(vinyl alcohol)/poly(ethylene glycol) produced bimodal open-celled structures with interconnected networks [39]. Poly(ɛ-caprolactone) resulted in structures with porosities between 60–80%, which represents a good potential for bone tissue engineering [40]. PLGA was combined with bioactive lipids for bone regenerative purposes [41], or PCL/PLGA blends [42], among others.

As far as the authors know, no previously published study has investigated scaffolds with this mixture of polymers, PLGA–PEDOT:PSS, generated by the supercritical foaming process presented herein. The objective of this study is to produce porous systems using a combination of non-conductive and conductive polymers for their potential application in tissue-repairing treatments. For this purpose, the authors evaluated the effects of certain key variables of the process, namely, temperature, pressure, and contact time, on the expansion factor, conductivity, mechanical strength, porosity, and the degradability of the final scaffolds.

## 2. Materials and Methods

### 2.1. Materials

PLGA (lactide:glycolide 75:25) (poly(lactic-co-glycolic acid)) with Mw 76,000–115,000 and PEDOT:PSS (Poly(2,3-dihydrothieno-1,4-dioxin)-poly(styrenesulfonate)) 3.0–4.0% in H_2_O and conductivity > 200 S/cm were purchased from Sigma–Aldrich (Spain). CO_2_ (99.8% purity) was supplied by Linde (Spain). Disodium-hydrogen phosphate (Na_2_HPO_4_) with 99.9% purity, potassium chloride (KCl) with 99.0% purity, potassium dihydrogen phosphate (KH_2_PO_4_) with 99.9% purity, and sodium chloride (NaCl) with 99.9% purity were purchased from Panreac Applychem (Barcelona, Spain).

### 2.2. Supercritical Foaming Procedure

**Figure 1:**
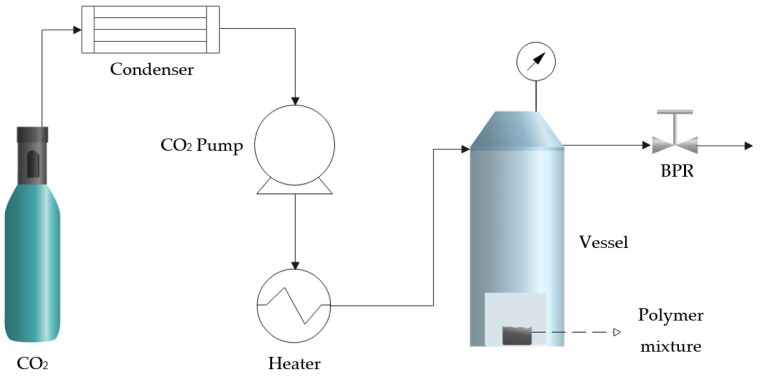
Flow diagram of the supercritical foaming equipment.

The scaffold foaming experiments were conducted at a pilot plant (TharProcess, Pittsburgh, PA, USA). A simplified diagram can be seen in Figure 1. The plant was equipped with a CO_2_ storage tank and a condenser to transfer the CO_2_ to the liquid state and to be able to pump it into the vessel (250 mL) with a high-pressure pump. Afterward, both the inlet CO_2_ and the interior of the vessel are heated by a heat exchanger and a heating jacket. Finally, the system was fitted with a micrometric valve (Back-Pressure Regulator—BPR) to regulate the depressurization rate and achieve the full venting of the CO_2_.

**Figure 2:**
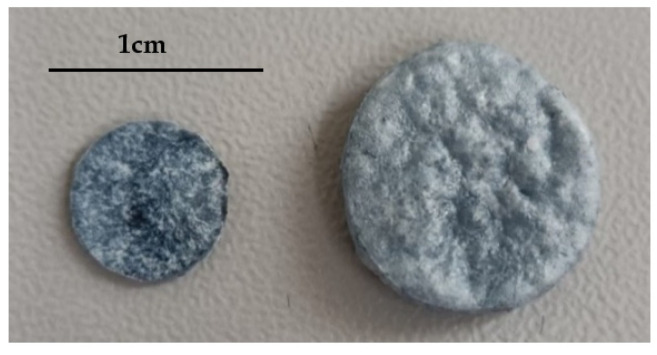
Photograph of a sample before (**left**) and after being subjected to the foaming process (**right**).

A total of 25 mg of PLGA 75:25 and 200 µL of PEDOT:PSS were mixed for each experiment and then shaped into a round tablet (30 mm^3^) by means of a tablet press machine. The tablets were placed in an aluminum foil holder and inserted into a high-pressure chamber. The system was then adjusted to the desired pressure and temperature, and CO_2_ was pumped in until supercritical conditions were reached. The polymeric mixture pellet was kept in contact with the supercritical phase for a specified amount of time to allow the CO_2_ to penetrate and cause polymer plasticization. After each specific saturation time, the system was depressurized at a controlled rate by means of the BPR. The foaming effect, i.e., the sample size and porosity increments, was achieved thanks to the rapid depressurization, and the PEDOT:PSS molecules were found to be evenly distributed throughout the entire tablet. The effect of temperature (30–60 °C), pressure (60–300 bar), and contact time (0.5–24 h) on the final porous structure was studied. Figure 2 shows one of the tablets used for the experiments before and after being subjected to the foaming process.

### 2.3. Scanning Electron Microscopy (SEM)

The morphology and pore diameter of the foamed scaffolds were examined by Scanning Electron Microscope Nova NanoSEM 450TM (Elecmi, Zaragoza, Spain) with 30 kV accelerating voltage. To improve the conductivity of samples in SEM and provide better image quality, a cross-section of each sample was coated with a 10 nm film of gold prior to analysis. The SEM images were used to calculate the pore diameter by Scion image software, measuring a minimum number of 300 pores for each experiment.

### 2.4. Polymer Distribution

Energy-dispersive X-ray spectroscopy (EDX) was used to analyze the chemical composition of different sections of the samples. The samples were directly deposited on carbon tape sections fixed on aluminum supports, and no gold coating was applied in order to avoid any interferences between sulfur and gold.

The Atomic Force Microscopy (AFM) images were obtained using a Bruker Dimension Icon microscope. A thin slice of the sample was mounted onto a metallic disc glued with double-sided tape, and, for the electrical measurements, a thin layer of silver paint was used to connect the sample surface to the metallic disc and the microscope stage. Given the fact that Young’s moduli of PEDOT:PSS and PLGA are of the order of a few GPa [43,44], for the nanomechanical measurements performed using the Quantitative Nanomechanics kit from Bruker, a Bruker RTESPA probe (nominal spring constant, k = 40 N/m, tip radius 8 nm) was selected since it is the one recommended for Young’s modulus ranging from 0.2 to 8.1 GPa. On the other hand, to obtain the surface potential maps by means of Kelvin Probe Force Microscopy (KPFM), a Bruker PFQNE-AL probe was used (k = 0.8 N/m, tip radius 5 nm). In this case, the work function of the tip was measured by using a gold/aluminum calibration sample (Bruker PFKPFM-SMPL) since the work function of gold can be averaged to 5.38 eV [45].

### 2.5. Expansion Factor of Samples

Volume variation of the different samples after processing with supercritical CO_2_ was quantified by the determination of the final expansion factor. The initial volume of each pellet could be directly measured thanks to its uniform surface and form, while its final volume was determined by submerging them into ethanol and following the liquid displacement method. The expansion factor was calculated according to the following expression:

(1) $$EF=\frac{Final\;volume}{Initial\;volume}$$

### 2.6. Estimated Porosity

The porosity of the formed scaffolds was determined by liquid displacement. Ethanol (96%) was chosen as the working fluid because it is capable of penetrating through the polymeric structure without any disruption. Porosity was determined by the rate between the void volume (the pores) and the total volume of the scaffolds. This estimation is based on Archimedes’ principle [46,47]. Thus, an estimation of porosity was calculated according to the following equation:

(2) $$Porosity\left(\%\right)=\frac{void\;volume}{Scaf\;fold\;volume}=\frac{V_{1}-V_{3}}{V_{2}-V_{3}}\cdot 100$$

where *V*_1_ is the initial volume of ethanol, *V*_2_ is the volume with the sample immersed till saturation, and *V*_3_ is the ethanol residual volume after sample removal.

### 2.7. Degradability Test in PBS

The degradability of the scaffolds was evaluated by their degradation in phosphate buffer saline (PBS) 0.01 M solution at 37 °C. The weight of the samples as a function of degradation time was calculated gravimetrically [47]. Excess PBS was removed by filter paper at each measurement. The assessments were taken every 2 days during the first 10 days of analysis and every 10 days during the following 40 days (50 days in total). The scaffolds’ mass remaining after 50 days were determined according to the following equation:

(3) $$Mass\;remaining\left(\%\right)=\frac{W_{2}}{W_{1}}\cdot 100$$

where *W*_1_ is the initial weight before the test and *W*_2_ is the final weight after 50 days in PBS.

### 2.8. Mechanical Properties

In order to study the mechanical endurance of the scaffolds, Young’s modulus (E) was determined by a compression test and was calculated as the slope of the elastic region in the stress–strain curve of the compression test [48]. The compression tests were carried out by means of an MTS Criterion C45 tester, where scaffolds were compressed to a total strain of 70% using a compression rate of 0.02 mm/s and a maximum load of 10 kN. The sizes of the samples were adjusted to 15 mm^3^ prior to analysis.

### 2.9. Conductive Properties

The electrical conductivity properties of the polymer scaffolds were measured by the two-probe method [49], using a resistance meter (TRMS Fluke 87-V). The samples were cut into 13 mm^2^-sized pieces. Each of the surfaces was put in contact with a copper sheet, which had a thickness of 250 µm. Electrical resistivity was measured using the 0–10 V voltage range. This analysis was carried out in duplicate.

## 3. Results and Discussion

In preliminary experiments with PLGA and PEDOT:PSS, a large amount of PEDOT:PSS outside the scaffold was detected in excess. Accordingly, the PLGA–PEDOT:PSS ratio was optimized to avoid this large excess of the PEDOT:PSS. Different amounts of conductive polymer were tested with respect to PLGA, which were 200, 400, and 600 µL. This represented a mass ratio of 1:8, 1:16, and 1:24, respectively. These previous experiments were carried out at average operating conditions, i.e., 200 bar pressure, 40 °C temperature, and 2 h contact time, where the foaming of PLGA is favored. Moreover, at 1:16 and 1:24 mass ratios, a greater amount of PEDOT:PSS was found outside the scaffold. The part of it that was found on its surface was easily detached. An amount of 200 µL of the conductive polymer was therefore used for the subsequent experiments, as this amount did not result in any excess polymer, and the scaffolds were reasonably homogeneous across their surface.

Under these conditions, the rest of the experiments were successfully carried out, and it was observed that the PLGA was foamed to a greater or lesser extent as a scaffold. On the other hand, the PEDOT:PSS was evenly distributed throughout their entire structure. In order to evaluate the reproducibility of the process, a replication (experiment 7) was performed. The similarity of the results from experiments 6 and 7 confirmed the reproducibility of the process. The effects of temperature, pressure, and contact time on the morphology, pore diameter, porosity, expansion factor, conductivity, and degradability were studied in order to establish a connection between the operating conditions and the scaffold properties and potential applications.

### 3.1. Polymer Distribution

**Figure 3:**
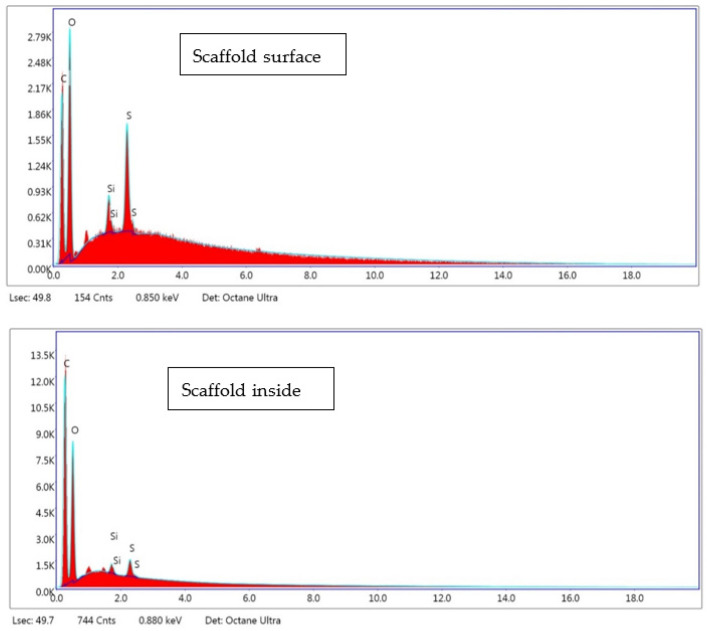
EDX spectra corresponding to composition on the surface and inside the scaffold (experiment 3).

A number of EDX analyses were carried out on different areas of the scaffolds (experiment 3) in order to identify any present PEDOT:PSS. As this polymer, unlike PLGA, contains sulfur, this compound was selected as the indicative of the PEDOT: PSS presence. An example of EDX spectra can be seen in Figure 3, where sulfur is clearly identifiable. Sulfur contents ranging from 0.8 up to 4.7 wt% on different surface areas and from 1.3 up to 2.6 wt% inside the scaffold were registered. It appears that there was a higher proportion of sulfur on the surface and, therefore, a higher amount of PEDOT: PSS. However, it should also be emphasized that in several areas within the scaffold, the amount of sulfur was noticeable. In line with EDX analyses, it was determined that PEDOT:PSS is located on the top and inside the scaffold. In order to discern if PEDOT was covering the scaffold surface as a film, AFM analyses were carried out for this scaffold.

**Figure 4:**
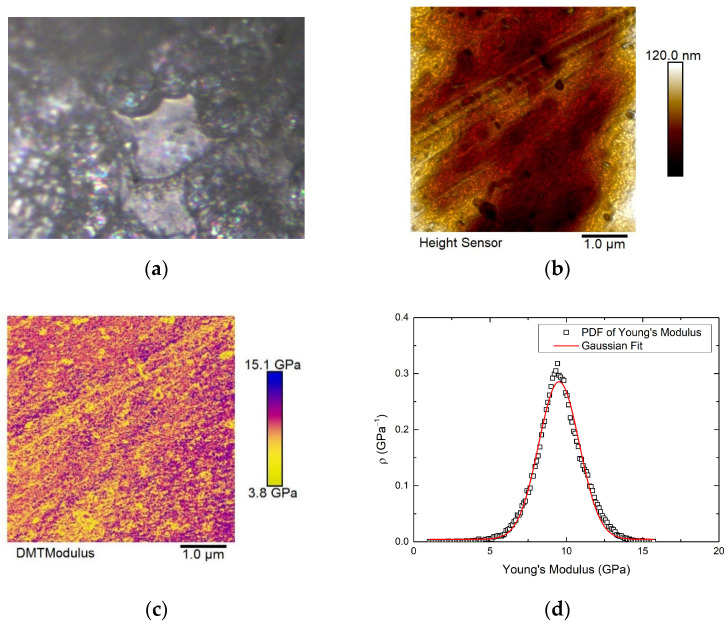
(**a**) Optical photograph of the flat area scanned for nanomechanical and electrical measurements. (**b**) Topography of a region of the sample (5 × 5 um scan). The minimum height data value has been shifted to zero. (**c**) Young’s modulus map of the area scanned in (**b**). (**d**) Probability density function of Young’s modulus values in (**c**).

Since the surface of the sample generally showed high roughness, a flat region of the sample surface was selected for nanomechanical and electrical measurements to reduce the interference of the purely topographic features on these parameters. Figure 4a shows the optical image of this flat area selected for the AFM scans, together with the topography of a 5 × 5 µm scanned area (b) and its corresponding Young’s modulus map, which looks quite homogeneous. Thus, the probability density function for Young’s modulus is shown in (d). These values were fitted to a gaussian function, and an average value of 9.5 GPa was obtained, which was close to the values previously reported for PEDOT:PSS and PLGA thin films [43,44].

Since PLGA shows similar Young’s modulus values to those of PEDOT:PSS [50], a different method is needed to demonstrate the presence of both polymers on the sample surface. PEDOT:PSS is a conducting polymer, and a large difference in work function is expected with respect to that of PLGA. The relationship between the work function of the AFM tip (χ*_tip_*), the work function of the sample surface (*χ_sample_*), and the contact potential difference (*V_CPD_*) between the tip and the sample is the following:

(4) $$V_{CPD}=\frac{\chi_{tip}-\chi_{sample}}{e}$$

where e is the elementary charge and *V_CPD_* is the parameter measured as surface potential in KPFM experiments. The KPFM probe was calibrated using a gold standard sample, and an average *V_CPD_* of 0.50 V was obtained for gold, whose work function value is 5.10 eV. Then, the work function of the tip was calculated as follows:

(5) $$\chi_{tip}=\chi_{Au}+e\cdot V_{CPD}=5.10\;\mathrm{eV}+\left(0.50\mathrm{eV}\right)=5.60\mathrm{eV}\;$$

Considering this calibrated value, the work function of the samples measured with this tip can be deduced from the following equation:

(6) $$\chi_{sample}=5.60\;\mathrm{eV}-{e\cdot V}_{CPD}$$

**Figure 5:**
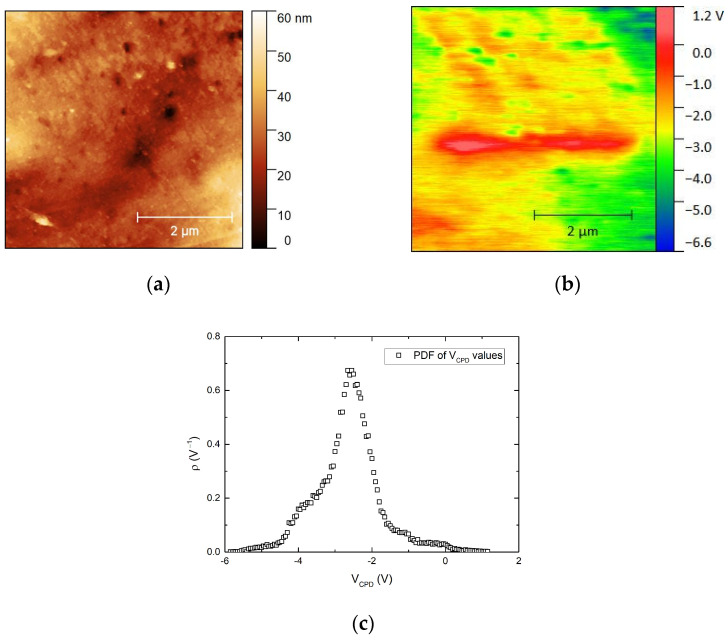
(**a**) Topography (5 × 5 µm scan) and (**b**) KPFM images. (**c**) Probability density function of the contact potential difference (VCPD) values mapped in (**b**).

Thus, Figure 5a shows the topography of another 5 × 5 µm area of the same flat region shown in Figure 4, together with the corresponding surface potential map (i.e., *V_CPD_* map) (Figure 5b), and the probability density function of *V_CPD_* values all over the scanned surface (Figure 5c). It may be readily deduced that the work function is not homogenous along the scanned area.

From these *V_CPD_* values, work function values ranging from 5.5 to 11.2 eV are deduced from Figure 5b. The reported value for the work function of PEDOT:PSS in thin films is 5.1 eV [51]. No reported values of the work function of PLGA have been found, but the most probable explanation for this result is that the surface sample is composed of a mixture of PLGA and PEDOT:PSS arranged in such a way that it is not possible to distinguish which regions are occupied by both polymers, given the lateral resolution of the KPFM technique (50–100 nm).

To confirm this, additional KPFM measurements were made in a pure PEDOT:PSS film and a pure PLGA pressed pellet, as shown in the Supplementary Information. The PEDOT:PSS film was deposited by spin coating using a 3% PEDOT:PSS aqueous solution onto a HOPG (highly oriented pyrolytic graphite) substrate. The work function deduced for this PEDOT:PSS film was 5.16 eV, which is very close to the previously reported value. However, for the pressed pellet of PLGA, the KPFM images showed that its *V_CPD_* values were so close to the lower limit of detection of the instrument (−10 V) that only in a few small areas of the sample *V_CPD_* values are within the available range, and its variation can be registered in the experiments. Its average value must be then beyond this −10 V limit. Even considering an average value of *V_CPD_* in PLGA of −10 V, its work function would be, according to this experiment, of the order of 15 eV at least. Then, the range of *V_CPD_* values found for the sample would necessarily imply that the surface of the sample is made of a combination of PEDOT:PSS and PLGA.

### 3.2. Effect of Temperature

**Figure 6:**
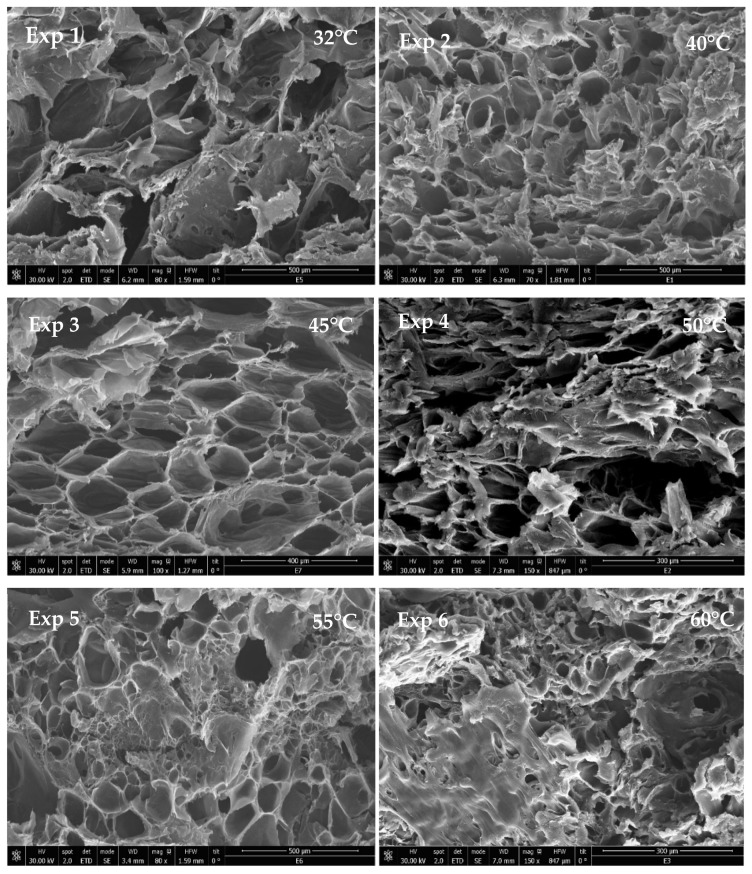
SEM images of formed scaffolds at 120 bar of pressure and 30 min of contact time at different operating temperatures.

The temperature was varied from 32 to 60 °C, keeping constant the rest of the parameters, as can be observed in Table 1. The morphology of the samples can be observed in Figure 6, where SEM images are shown. The formed scaffolds are based on PLGA, but PEDOT:PSS did not appear as particles. If both the AFM analyses and [18,24] are taken into account, it follows that PEDOT:PSS is well integrated, and it is the covering part (i.e., film) of the PLGA surface.

It can be observed in Table 1 that pore diameter increased significantly at the lowest temperature tested. Then, as the temperature was increased, the pore diameter fluctuated until the temperature reached 60 °C, after which smaller pore diameters were obtained. The same trend was observed with regard to standard deviation, so lower-standard deviations were registered at higher temperatures. In any case, and according to the SEM images, the highest geometry of the pores was achieved at 45 °C. With regard to porosity rate, it seemed to decrease at higher temperatures since their smaller pore diameters resulted in lower porosity rates, while the scaffolds with larger pores also had greater porosity rates. Therefore, the total porosity of the scaffolds seems to be more dependent on pore size rather than on pore number, even if the number of small pores was greater in those scaffolds that had been processed under higher temperatures. In this way, when the temperature is increased while pressure remains constant, the density of CO_2_ decreases, and so does its solvent power. On the other hand, at temperatures above the glass transition of PLGA (45–47 °C) [52], the polymer reaches its rubber state, and CO_2_ diffuses easily into the polymer matrix.

Different research studies in the literature deal with the formation of PLGA porous scaffolds using other techniques, such as 3D printing, electrospinning, or electrospun. This last one produced larger size pores. On the other hand, supercritical foaming generated macropores between 150–500 µm [53,54,55,56] with a larger pore size distribution and also allowed better control of this parameter just by implementing minor changes in its key variables.

Thus, the expansion factor proved to be strongly influenced by temperature, so greater expansion factors were achieved at lower temperatures. At constant pressure, an increase in temperature reduces the density of CO_2_ and its ability to solubilize and penetrate into the polymer matrix. This poorer soaking of CO_2_ into the polymer results in a lower number of bubbles and, therefore, a lower volume growth. The same behavior had been observed in a previous work, where PLGA was foamed while simultaneously impregnated with rutin [36].

With regard to the conductivity levels measured (Table 1), it can be observed that the higher the expansion factor, the lower the conductivity. The lower conductivity of the scaffolds [33] can be explained by the higher porosity resulting in a higher amount of trapped air and by a higher disorder between the PEDOT:PSS molecules. The conductive scaffolds prepared in this work have useful electrical characteristics, achieving a maximum conductivity of 1.6 × 10^−5^ S/cm, and therefore, they may be a suitable candidate for nerve, bone, vascular, or cardiac muscle tissue replacements that generally need to conduct electrical signals [57,58].

**Figure 7:**
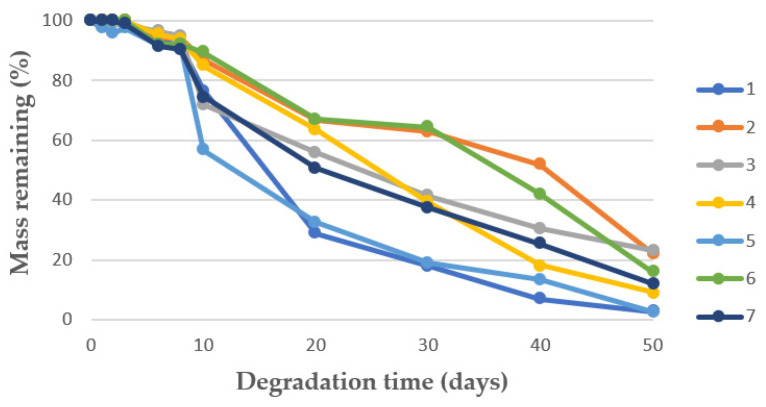
Mass remaining vs. degradation time in PBS solution of the scaffolds produced under different temperature levels.

The degradability of the scaffolds, as a function of their weight loss, was measured for fifty days, as can be seen in Figure 7. The mass remaining at the end of the assay can be seen in Table 1. This factor is crucial to find out the suitability of the scaffolds for tissue engineering purposes. According to the data in Table 1, the scaffolds that were generated in experiments 2 (40 °C) and 3 (45 °C) presented the lowest degradation, with a final mass remaining or around 22–23 wt.%. Contrarily, the scaffolds that were most degraded had been produced at 32 and 50 °C. Nevertheless, no clear correlation could be established between temperature and degradability. During the first stages of the degrading process, a small number of glycolic and lactic units are hydrolyzed and released. The length of this first stage of degradation depends on each polymer’s lactic content, so that the greater the content, the longer this first stage is. In all the cases, a second degrading phase started after approximately ten days. This can be explained by the fact that during the period of 50 days, the medium becomes very acidic due to the autocatalyzed PLGA hydrolysis reaction, which increases the degradation rate [59,60].

### 3.3. Effect of Pressure

**Figure 8:**
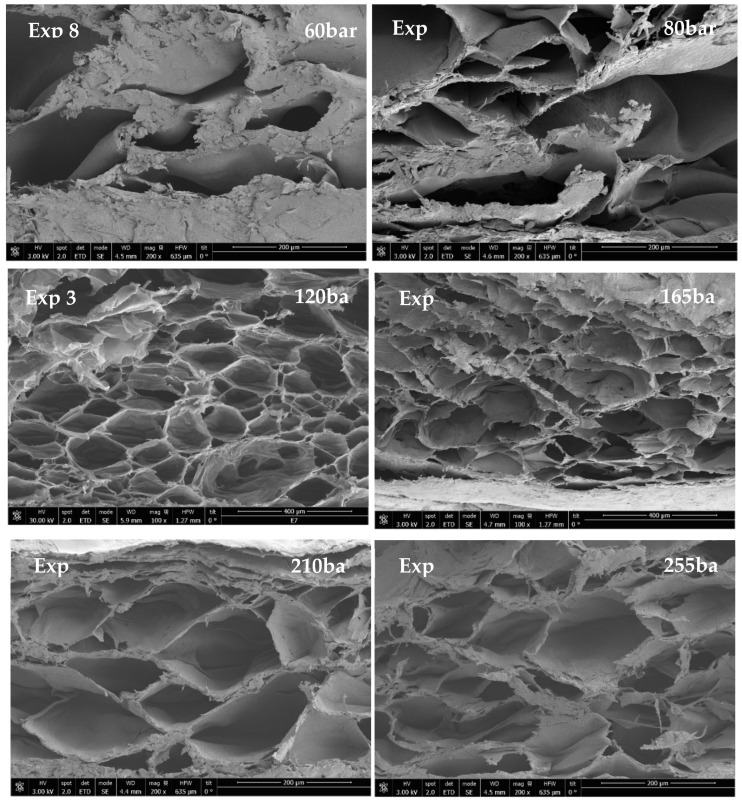
SEM images of formed scaffolds at 45 °C of temperature and 30 min of contact time at different operating pressures.

In order to evaluate the effect of pressure on the scaffolds’ properties, the temperature (45 °C) and the contact time (0.5 h) were held constant, and different pressure levels were tested. Figure 8 displays the SEM images of the scaffolds. Foaming under subcritical conditions (experiment 8) was also evaluated, and the greatest average pore diameter, as well as the lowest porosity rate, were recorded. The size of the polymers’ pores is correlated with the number of CO_2_ bubbles that are produced according to the different experimental conditions. Thus, it can be observed that pore diameters are much greater when subcritical pressure is used for the foaming process, which is possibly due to the particular expansion profile that foam exhibits under these conditions [61].

In this sense, when pressure rises over CO_2_ critical pressure, the average diameter of the pores obtained is smaller. This is due to the higher solubility of the CO_2_ in the polymeric matrix, which decreases the nucleation energy barrier [46]. Similar behaviors have been observed when foaming other polymers [62,63,64]. Not only did the average pore diameter decrease with higher pressure levels, but pore uniformity was also enhanced. This can be observed in Table 2, where experiment 12 (255 bar) produced the scaffold with the smallest pore average diameter and the most regular pores.

Throughout all the experiments, pore diameters generally between 30 and 200 nm and porosity rates up to 50% were obtained. According to the literature, this would allow the use of the scaffolding for a diversity of applications depending on their actual porosity. Their applications could include osteogenesis (150–200 nm pore diameter, 35% porosity) [65], skin regeneration (20–125 nm pore diameter) [66], or smooth muscle cell differentiation (20–250 nm pore diameter) [67], among others.

The porosity of the scaffolds increased as the pressure was escalated up to 165 bar. Thus, as pressure was increased, more CO_2_ was absorbed by the polymer matrix, resulting in more CO_2_ bubbles and greater porosity. However, at pressures above 165 bar and up to 255 bar, the porosity rate dropped. This occurs because higher pressure increases the diffusivity of the CO_2_ into the polymer up to a point where pores cannot withstand the excessive pressure, and they collapse [64,65,66]. The expansion factor proved to be highly dependent on porosity rate so that it followed the same trend, i.e., it increased with pressure until 165 bar and then went down in the range from 165 to 255 bar.

Conductivity seemed to be inversely correlated with the expansion factor, i.e., as the pressure and expansion factor were increased, the conductivity was reduced. Hence, the scaffolds were more conductive when produced at lower pressure levels (Table 2). As above said, the larger population of voids in the polymer foams affected the electrical conductivity of the scaffolds and resulted in disorder between PEDOT:PSS molecules. For this reason, the scaffolds that exhibited the highest conductivity were those that had been produced under the pressure range between 165 and 255 bar, which resulted in a lower expansion factor. Overall, the conductivity was higher when the growth of the polymer was lower, thus avoiding disruption between PEDOT:PSS molecules along the polymer structure.

**Figure 9:**
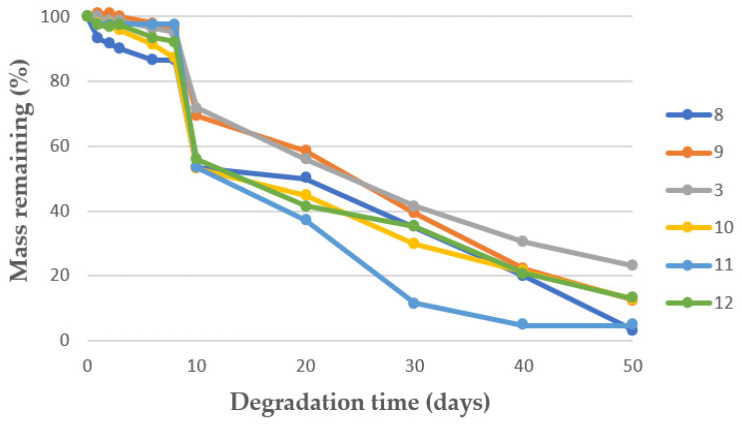
Mass remaining vs. degradation time in PBS solution of the scaffolds produced under different pressure levels.

With regard to degradability, it can be observed from Table 2, and Figure 9 that experiments 8 and 11 had the highest degradability rates, and experiment 3 had the lowest one, but no correlation with pressure could be identified. It seems that the degradation around the tenth day resulting from the hydrolysis and acidification of the media does not take place with the same intensity in every scaffold. This could be explained by the different distribution of the PEDOT:PSS molecules inside the PLGA structure.

### 3.4. Effect of Contact Time

**Figure 10:**
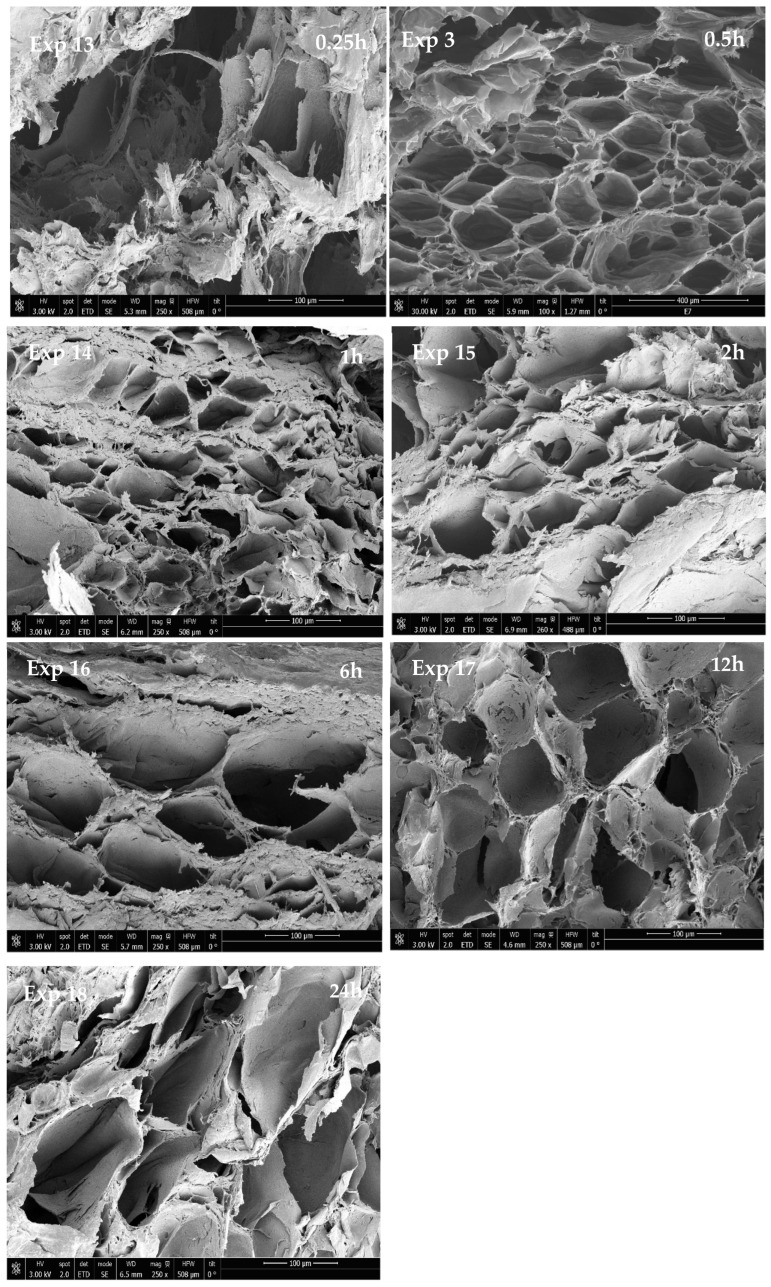
SEM images of formed scaffolds at 120 bar of pressure and 45 °C of temperature at different contact times.

In order to determine if there was any correlation between the contact time of the scCO_2_ with the polymer and the obtained scaffold properties, a series of experiments were performed (see data in Table 3). The morphology of the scaffolds resulting from this set of experiments can be observed in Figure 10.

According to the images, 0.25 h contact time does not seem to be long enough to produce a scaffold structure. The foams obtained under these conditions presented poor porosity, with a large pore average diameter and widely variable shape. However, despite this lack of uniformity, these structures exhibited high conductivity values. This was probably explained by their low expansion factor, which contributed to avoiding the disruption of the PEDOT:PSS molecules and, in turn, favored electrical conductivity.

**Figure 11:**
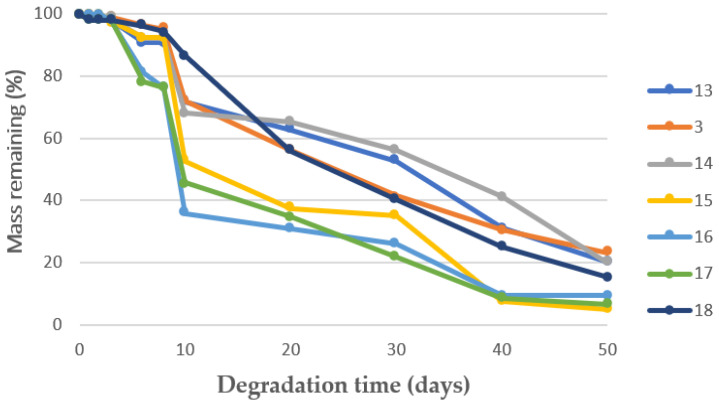
Mass remaining vs. degradation time in PBS solution of the scaffolds obtained after varying contact times.

Contact time does not seem to have a clear effect on average pore diameter. Nevertheless, the maximum porosity rate was registered after 0.5 h contact time. The same trend was observed with respect to the expansion factor, which reached its maximum value also after 0.5h contact time. This was in consonance with CO_2_ sorption, which did not seem to increase with contact times longer than 0.5 h. Conductivity, on the other hand, remained unaltered regardless of the contact time used. Finally, it seems that the scaffolds exhibited higher resistance to degradation in PBS when they were produced using shorter contact times, as can be seen in Table 3 and Figure 11. However, the scaffolds that had been produced after 24 h contact time (experiment 18) also registered higher mass remaining than some of the scaffolds obtained after shorter contact times. Therefore, no definite trends could be established.

### 3.5. Mechanical Properties

The mechanical properties of several of the scaffolds that had been produced were evaluated to determine the influence of temperature, pressure, and contact time on the toughness of the scaffolds. This is an essential factor to take into account if the scaffolds are intended to be used as cell-supporting matrices for tissue regeneration.

Table 4 summarizes the various measured properties and Young’s modulus. The modulus has been determined by the slope of the linear region of the stress–strain curves, which indicates the material’s elastic behavior. The resistance of body tissues ranges between 2 and 6000 MPa depending on the type of tissue so that these values would correspond to a mature bone or to fibrous tissue, respectively [67,68,69,70].

The scaffolds obtained by experiment 11 exhibited the highest Young’s modulus and, therefore, the best mechanical properties. The scaffolds from experiments 10 and 14, which had been produced at 45 °C, also registered high modulus. A priori, the expansion factor should be inversely related to the toughness of the material. In this sense, the scaffold from experiment 11, with the lowest expansion factor, met this trend. Likewise, the scaffold obtained from experiment 1 and presenting the highest expansion factor, had a low Young’s modulus. However, the scaffold from experiment 4, with the same expansion factor as the latter, exhibited the poorest mechanical resistance. In the rest of the cases, no relationship between the expansion factor and mechanical resistance could be established. This was probably due to the specific distribution of the PEDOT:PSS molecules within the polymer structure, which might confer certain fragility to the scaffolds.

Most of the tests that have been performed revealed an improvement in the mechanical strength of the PLGA foamed. This is in line with the results reported in the literature when this material was foamed using other techniques, such as 3D printing, and where Young’s modulus compression tests registered values between 1.5 and 3.7 MPa [53].

## 4. Conclusions

PLGA–PEDOT:PSS scaffolds have been produced by means of a reproducible super-critical foaming process. According to EDX analyses, PEDOT:PSS was distributed on the surfaces and the inside of the PLGA scaffolds. It has also been confirmed, via the AFM analyses, that the surface of the scaffolds obtained was formed by a combination of PEDOT:PSS and PLGA. The effects of temperature, pressure, and contact time on the morphology, average pore diameter, porosity rate, expansion factor, conductivity, and degradability have been evaluated. This evaluation has allowed us to determine the trends that should be taken into account for the final scaffolds to exhibit the desirable properties for their intended applications. Thus, the expansion factor of the foams was strongly influenced by temperature, so that the lower the temperature, the higher the expansion factor. The foams’ conductivity, on the contrary, became lower as their expansion factor increased. Temperature presented an inverse relation with porosity so that the largest pore diameters were obtained at the lowest temperatures tested. Although the porosity of the scaffolds went slightly down as the temperature increased, their maximum porosity was reached at 165 bar regardless of the temperature. At pressure levels above 165 bar, porosity decreased, possibly due to the collapsing of the pores because of the excessive pressure. Moreover, smaller and more uniform pore diameters were obtained under the highest pressure levels tested.

Contact times shorter than thirty minutes did not seem to be adequate to form a proper scaffold structure, even if the foams obtained after thirty minutes exhibited a higher conductivity. The maximum porosity and expansion factors were obtained after a thirty-minute contact time, while longer times did not improve the sorption of the CO_2,_ neither did the porosity rate nor the expansion of the foams. In any case, no clear contact time trends could be established, even if the scaffolds that had been produced with shorter times presented a greater resistance to degradation and a greater mass remaining (22–23 wt%). In this sense, the scaffolds produced at 210 bar, 45 °C, and using a thirty-minute contact time had the greatest mechanical resistance but the lowest expansion factor. It should be added that under subcritical conditions, the foams presented the largest average pore diameter and the lowest porosity rate.

Using supercritical CO_2_ to produce therapeutic scaffolds has allowed us to obtain foam structures with a wide range of average pore diameters (30–200 nm), porosity rates (up to 50%), and conductivity (up to 1.0 × 10^−5^ S/cm). This versatility of options corroborates the suitability of the final scaffolds for diverse medical treatments, such as the regeneration of nerve, bone, vascular or cardiac muscle tissues.

## Figures and Tables

**Table 1:** Supercritical foaming experiments to evaluate the influence of temperature on the process.

Experiments	t (t)	P (bar)	T (°c)	Pore Diameter (nm)	Porosity (%)	Expansion Factor	Conductivity (S/cm)	Mass Remaining (%)
1	0.5	120	32	246.4 ± 77.7	51.5 ± 1.4	10.7	6.3 × 10^−7^	2.7 ± 1.2
2			40	107.5 ± 41.4	42.9 ± 0.1	5.5	3 × 10^−6^	22.2 ± 3.5
3			45	127.8 ± 52.0	47.7 ± 2.3	6.2	1.2 × 10^−6^	23.2 ± 9.7
4			50	86.5 ± 76.0	40.8 ± 0.8	5.1	1.9 × 10^−6^	9.1 ± 3.5
5			55	113.5 ± 46.2	42.3 ± 0.6	4.9	4.5 × 10^−6^	2.7 ± 2.5
6			60	32.4 ± 16.0	29.2 ± 4.1	1.4	1.6 × 10^−5^	15.8 ± 10.1
7			60	31.8 ± 8.6	29.1 ± 4.0	1.4	3.1 × 10^−5^	12.0 ± 1.4

**Table 2:** Supercritical foaming experiments to evaluate the influence of pressure on the process.

Experiments	t (h)	T (°C)	P (bar)	Pore Diameter (nm)	Porosity (%)	Expansion Factor	Conductivity (S/cm)	Mass Remaining (%)
8	0.5	45	60	331.3 ± 345.8	29.2 ± 4.2	4.9	2.6 × 10^−6^	3.3 ± 1.4
9			80	114.5 ± 100.9	40.8 ± 0.8	5.9	1.1 × 10^−6^	12.5 ± 1.1
3			120	127.8 ± 52.0	47.7 ± 2.3	6.2	1.2 × 10^−6^	23.2 ± 6.9
10			165	116.9 ± 71.1	50.0 ± 0.1	7.4	2.2 × 10^−7^	12.8 ± 4.6
11			210	117.4 ± 49.7	42.2 ± 2.2	5.1	1.1 × 10^−6^	4.7 ± 1.1
12			255	96.2 ± 43.4	33.3 ± 0.1	3.4	3.8 × 10^−6^	13.0 ± 8.7

**Table 3:** Supercritical foaming experiments to evaluate the influence of contact time on the process.

Experiments	P (bar)	T (°C)	t (h)	Pore Diameter (nm)	Porosity (%)	Expansion Factor	Conductivity (S/cm)	Mass Remaining (%)
13	120	45	0.25	213.8 ± 151.9	30.9 ± 2.4	2.4	1.0 × 10^−5^	20.2 ± 2.4
3			0.5	127.8 ± 52.0	47.7 ± 2.3	6.2	1.2 × 10^−6^	23.2 ± 2.4
14			1	44.5 ± 21.4	50.0 ± 0.1	6.0	5.2 × 10^−7^	20.0 ± 5.9
15			2	61.1 ± 30.0	38.1 ± 4.8	5.4	1.6 × 10^−6^	5.1 ± 4.3
16			8	94.5 ± 34.4	53.6 ± 3.6	6.2	7.6 × 10^−7^	9.5 ± 4.1
17			15	97.4 ± 22.0	50.1 ± 0.1	7.7	3.5 × 10^−7^	6.5 ± 1.4
18			24	103.7 ± 37.5	36.7 ± 3.3	4.9	5.5 × 10^−6^	15.4 ± 5.7

**Table 4:** Summary of the data, including Young’s modulus, that corresponds to the scaffolds obtained from specific experiments.

Experiment	P (bar)	T (°C)	t (h)	PoreD (nm)	Porosity (%)	EF *	MR * (%)	C * (S/cm)	E (MPa)
1	120	32	0.5	246.4 ± 77.7	51.5 ± 1.4	10.7	2.7 ± 1.2	6.3 × 10^−7^	1.3
3	120	45	0.5	127.8 ± 52.0	47.7 ± 2.3	6.2	23.2 ± 9.7	1.2 × 10^−6^	1.2
4	120	50	0.5	86.5 ± 76.0	40.8 ± 0.8	5.1	9.1 ± 3.5	1.9 × 10^−6^	0.8
9	80	45	0.5	114.5 ± 100.9	40.8 ± 0.8	5.9	12.5 ± 1.1	1.1 × 10^−6^	2.2
10	165	45	0.5	116.9 ± 71.1	50.0 ± 0.1	7.4	12.8 ± 4.6	2.2 × 10^−7^	11.3
11	210	45	0.5	117.4 ± 49.7	42.2 ± 2.2	5.1	4.7 ± 1.1	1.1 × 10^−6^	13.6
14	120	45	1	44.5 ± 21.4	50.0 ± 0.1	6.0	20.0 ± 5.9	5.2 × 10^−7^	8.9
16	120	45	8	94.5 ± 34.4	53.6 ± 3.6	6.2	9.5 ± 4.1	7.6 × 10^−7^	3.3
17	120	45	15	97.4 ± 22.0	50.1 ± 0.1	7.7	6.5 ± 1.4	3.5 × 10^−7^	1.3

* EF = Expansion Factor. * MR = Mass Remaining. * C = Conductivity.

## Data Availability

Not applicable.

## Supplementary Materials

The following supporting information can be downloaded at https://www.mdpi.com/article/10.3390/ma16062441/s1, Figure S1. Pristine PEDOT:PSS thin film deposited by spin coating on HOPG. Figure S2. AFM.1. Pressed pellet of PLGA.
