# Supplementary material for: Formation of PLGA–PEDOT: PSS Conductive Scaffolds by Supercritical Foaming
Source: Materials (Basel). 2023 Mar 18;16(6):2441. doi: 10.3390/ma16062441 (PMC10057315; doi:10.3390/ma16062441)
Supplement: Supplementary file 1 [file materials-16-02441-s001.zip › materials-2138890-supplementary.pdf]

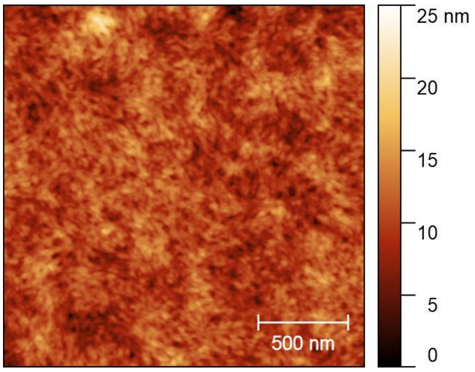

(a)

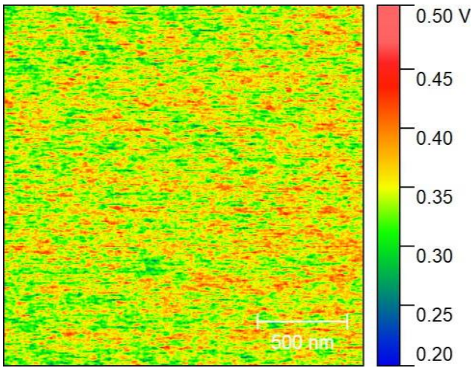

(b)

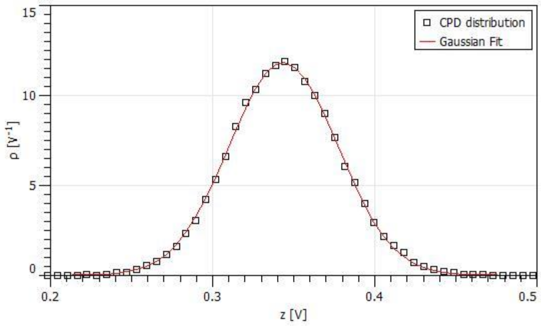

(c)

Figure Supplementary S1. Pristine PEDOT:PSS thin film deposited by spin coating on HOPG. (a) Topography (2x2 μm scan) and (b) KPFM images. (c) Distribution of CPD values in (b).

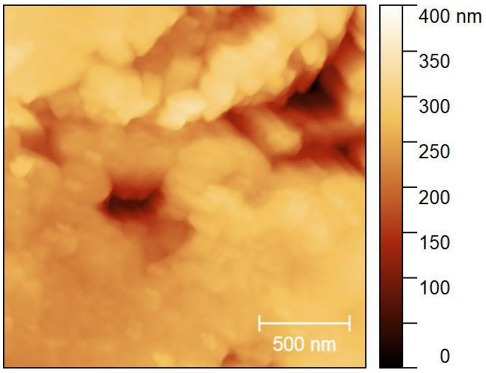

(a)

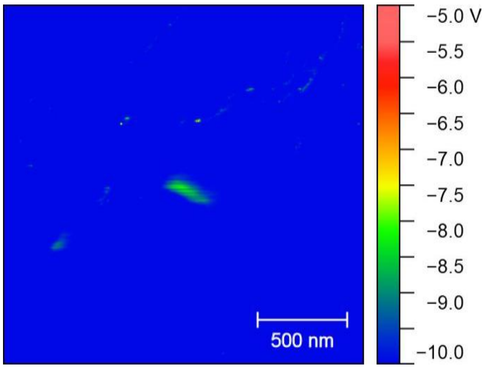

(b)

Figure Supplementary S2.AFM.1.Pressed pellet of PLGA. (a) Topography (2x2 μm scan) and (b) KPFM images.
